# Dissecting the Role of Disturbed ER-Golgi Trafficking in Antivirals and Alcohol Abuse-Induced Pathogenesis of Liver Disorders

**DOI:** 10.21767/2471-853X.100054

**Published:** 2017-09-26

**Authors:** Cheng Ji

**Affiliations:** Department of Medicine, Keck School of Medicine of USC, Los Angeles, CA, USA

**Keywords:** Alcohol abuse, HIV, AIDS, ER, Golgi apparatus, Liver disorders

## Abstract

Antiviral drugs and alcohol abuse-induced organelle stresses have been linked to many disorders and the underlying molecular mechanisms are under intense investigations. This brief review communicates emerging evidence and research trends on how certain antivirals and alcohol affect ER-Golgi trafficking, which potentially impacts the function and integrity of the Golgi apparatus contributing to endoplasmic reticulum stress and cellular injury.

## Introduction

Globally, there are 36.7 million people living with HIV and approximately 1.8 million acquire HIV infection every year [1]. In the United States, 1.2 million people are living with HIV infection and half million with AIDS have died since the HIV epidemic began in the 1980’s [1–3]. The situation demands development of antiretroviral drugs and access to antiretroviral therapies. While anti-HIV vaccines or genome editing are yet to be developed and put into practice, antiretroviral agents are effective [4–6]. For instance, HIV protease inhibitors (HIV PIs) that inhibit HIV proteinase or protease are used in the highly active effective antiretroviral therapies (HAART) and nucleic acid-based anti-HIV compounds are developed for further inhibition of intracellular viral targets. The quality of life of HIV/AIDS patients under anti-HIV therapies is improved significantly. However, there are numerous reports indicating that some antivirals singly or in combination increase the risk of comorbidities [7–9]. While some side effects of anti-HIV drugs are manageable, some can be very serious and fatal. For instance, hepatic injuries have emerged as the major non-AIDS–related cause of death among HIV/AIDS patients [8,10]. The risk of drug side effects gets worse as nearly half of the HIV-infected patients abuse/consume alcohol, which not only impairs patients’ adherence to HAART but also worsens anti-HIV drug-induced hepatotoxicity leading to greater morbidity and mortality [11,12]. Nonhazardous alcohol use of less than five standard drinks (equivalent to 5 × 14 g of pure alcohol) once a week can reduce survival of HIV/AIDS patients by one year and daily hazardous use of five or more standard drinks per day reduces survival by more than six years [11–13]. Increase in liver injuries such as liver fibrosis or cirrhosis are often seen at all levels of alcohol exposure [8,10,11]. To be even complicated, there are 10 million HIV/AIDS patients worldwide co-infected with hepatitis viruses and/or tuberculosis (TB). Additional drugs against the co-infections and/or drug abuse could further increase the severity of the hepatoxicities [8,14,15]. Therefore, it is of importance to dissect pathogenic mechanisms underlying the hepatoxicity caused by the drugs combined with alcohol consumption/abuse, which would provide basis for a better management of AIDS patients suffering from liver disease.

## Organelle Stress in Antivirals and Alcohol-Induced Liver Disorders

There are a few potential mechanisms underlying the alcohol and drug-induced hepatotoxicity/liver injury: (1) direct intrinsic toxicities from individual drugs [16]; (2) idiosyncratic hypersensitivity reactions [16]; (3) aberrant immune activities [16,17]; (4) metabolic abnormality and cellular stress response [18]. The intrinsic toxicity occurs dose-dependently at sub-lethal doses and can be influenced by environmental and genetic sensitivity factors. The idiosyncratic reactions occur in a minority of patients without obvious relationship to drug dose or time of onset. The innate and adaptive immune responses are generally known to be involved in the liver toxicity. Detailed characteristics of these potential mechanisms are not discussed in this focused review. The stress and metabolic abnormality mechanism is the most significant and relevant as cellular stress responses in the liver may generate danger signals which co-stimulate the immune system, and alcohol and majority of the anti-HIV drugs are metabolized in the liver by the cytochrome P450 enzyme system, which is bound to interfere with the metabolism and the antiviral therapies in AIDS patients. In fact, alcohol as “the first hit” is reported to affect hepatic CYP activities that metabolize protease inhibitors [18–20]. Both alcohol and certain antiviral drugs induce organelle stress, such as the induction of unfolded protein accumulation in the endoplasmic reticulum (ER) resulting in ER stress and cell death [20,21]. Anti-HIV drugs such as ritonavir (RTV), indinavir (IDV), lopinavir (LPV) or atazanavir (ATZ) have been reported to induce ER stress in hepatic cells [22]. We initially discovered that alcohol induced ER stress in the liver of animal models [23]. The ER stress normally triggers protective unfolded protein response (UPR), which involves three ER stress sensors: IRE1 (inositol requiring enzyme 1), PERK (PKR-like ER kinase) and ATF6 (activating transcription factor), to restore ER homeostasis and minimize injuries. However, prolonged UPR such as under conditions of chronic alcohol consumption and/or long-term anti-HIV therapies induces fat accumulation, inflammation and cell death, which are well documented to lead to development of hepatic steatosis, fibrosis and cirrhosis [20–25]. Because the ER stress response is a major pathogenesis mechanism, xenobiotics to ensure proper ER homeostasis have been developed and tested in a variety of *in vitro* and *in vivo* model systems. They include: molecular chaperones such as sodium 4-phenylbutyrate (PBA) and taurine conjugated ursodeoxycholic acid (TUDCA) that increase the ER protein-folding capacity; and compounds such as antioxidants, autophagy inducers, and UPR enhancers (e.g. salubrinal/guana benz, trans-4,5-dihydroxy-1,2-dithiane (DTTox), and valproate) that either increase expression of protein chaperones or enhance the protective UPR pathways [21,25,26]. However, the xenobiotics for ER homeostasis restoration resulted in partial protective effects, suggesting that although the ER stress is involved in the alcohol and drug-induced liver injuries, there are other cellular targets that either contribute or are upstream of the ER stress response.

The organelle that is closely associated with the ER is the Golgi apparatus. The Golgi is part of the cellular endomembrane system, in which secretory and membrane proteins from the ER receive various modifications such as glycosylation, phosphorylation and sulfation and are then packaged into membrane-bound vesicles before trafficking to their destinations. Similar to the ER, the structure and capacity of the Golgi can fluctuate according to physiological demands or pathological stress conditions. When the protein load and modifications exceed the Golgi capacity there is a Golgi stress response (referred to as GSR) to increase its capacity [20,27]. Impaired GSR can cause cellular injuries directly or indirectly through the ER stress. There are a few factors that may regulate GSR. The first is TFE3 (a basic-helix-loop-helix type transcription factor) [28], which regulates transcriptional activation of genes encoding vesicular trafficking components or Golgi residents such as RAB20, syntaxin 3A (STX3A), protein 60 (GCP60), GM130, giantin, sialyltransferase, fucosyltransferase and glycosylation enzymes. Translocation of TFE3 into the nucleus depends on the status of its phosphorylation. Upon Golgi stress, TFE3 is dephosphorylated and translocated into the nucleus where it activates the GSR genes. The second is the CREB3-ARF4 pathway involving both ER and Golgi [29]. CREB3 is a basic leucine zipper-containing transcription factor that resides in the ER membrane. ARF4 (ADP-ribosylation factor 4) is a member of the small GTPase family that regulates Golgi-to-ER vesicular trafficking. CREB3 is activated via proteolysis and upregulates the transcription of ARF4 in Brefeldin A (BFA) treated cells that are under both ER and Golgi stresses. BFA is known to inhibit the function of several guanine nucleotide exchange factors (GEFs) and blocks GEF-mediated Golgi-to-ER trafficking [30]. The third pathway involves an ER chaperone, HSP47. HSP47 may protect cells from the Golgi stress as expression of HSP47 is increased under the Golgi stress and suppression of HSP47 by siRNA resulted in fragmentation of the Golgi apparatus and cell death [31].

Alcohol consumption/abuse has long been known to induce ultrastructural changes in the Golgi. In man and animal, chronic *alcohol* feeding with nutritionally adequate diets induced *ultrastructural* abnormalities of the *intestinal* epithelial cells, mammary cells, hepatocytes, neurons, and glandular epithelium cells [32–38]. In addition to the morphological changes, there is evidence for alcohol-induced functional and metabolic changes. Chronic alcohol exposure affects the ER-Golgi trafficking in neuronal dendrites [39]. Alcohol alters glycosyl transferase activity in the Golgi of liver cells [40]. Acute ethanol intoxication interferes with various steps of protein glycosylation at the Golgi of rat liver [41]. Further, accumulations of lipid and carbohydrates and decreased terminal glycosylation were observed in the Golgi of isolated hepatocytes treated with alcohol, which was associated with production of anticytoplasmic autoantibodies. Significantly, high titers of anti-Golgi antibodies were detected in human alcoholics with hepatocellular carcinoma (HCC) [42].

There is also evidence that either viral infection or antiviral drugs induce the Golgi stress. For instance, hepatitis C (HCV) replication requires the guanine nucleotide exchange factor 1 (GBF1) and its effector ADP ribosylation factor 1 (Arf1) that are known to regulate Golgi membrane trafficking and organelle structure in the secretory pathway. HCV infection is reported to result in Golgi fragmentations [43]. On the other hand, pegylated interferon (Peg-IFN) plus ribavirin, the standard therapy for HCV, induces anti-Golgi antibodies, which is associated with liver injury in patients [44].

## Emerging Evidence and Hypothesis of Disrupted ER-Golgi Trafficking in Drugs and Alcohol-Induced Liver Pathogenesis

**Figure 1:**
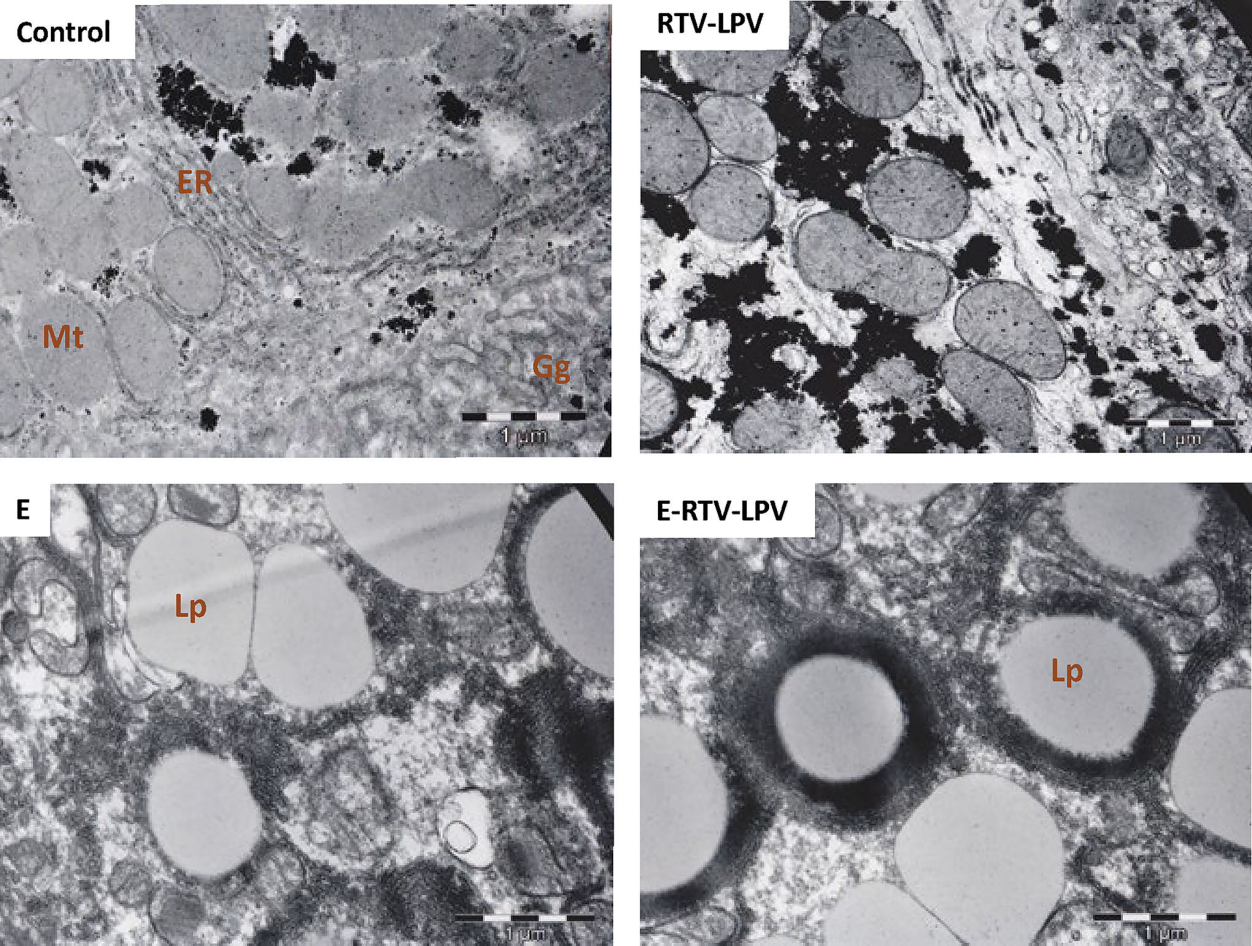
Electron microscope images demonstrating damages of the Golgi apparatus and endoplasmic reticulum of hepatocytes from animal models fed alcohol (E) and anti-HIV protease inhibitor ritonavir (RTV) and lopinavir (LPV) for one month. Gg: Golgi Apparatus; ER: Endoplasmic Reticulum; MT: Mitochondria; Lp: Lipid Droplets

Synergistic or additive effects of antivirals combined with alcohol on liver injury have been observed recently [8,18,45,46]. Chronic alcohol feeding of animal models resulted in fat accumulation, which is associated with distorted morphologies of the ER and Golgi organelles visible under electron microscopy (Figure 1). Treating the animals with a standard regimen for HIV-infected patients (i.e., ritonavir boosted lopinavir) induced moderate dilatation of the ER and dispersed Golgi apparatus. Combination treatments with alcohol and the drugs induced severe damages to the hepatocytes. Neither the ER nor the Golgi was identifiable and rare myelin-like structures surrounding giant lipid droplets were observed for the first time in the liver cells from treated animals. In contrast, the hepatocytes from control animals had lamella ER and normal Golgi apparatus.

**Figure 2:**
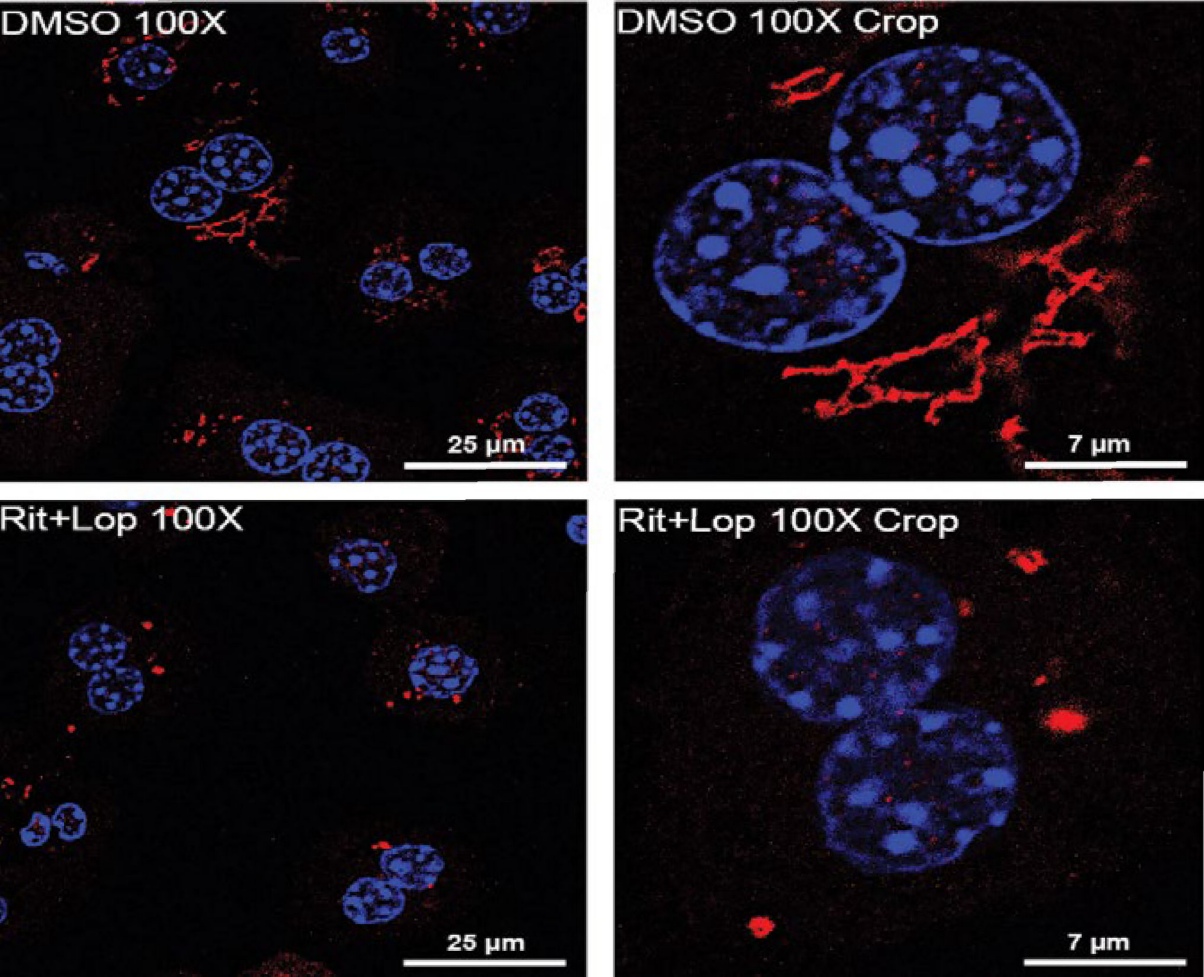
Confocal images of primary hepatocytes treated with vehicle DMSO versus ritonavir (RIT) and lopinavir (LOP). Fragmented Golgi apparatus can be seen in the liver cells treated with the anti-HIV drugs

In pursuing molecular details of the alcohol and drug-induced organelle stress response, we found that the three canonical UPR branches, IRE, PERK and ATF6 were differentially expressed in HepG2 or primary hepatocytes in response to the RTV boosted LPV treatment [18,46]. The ATF6 branch as well as genes/factors downstream of the ATF6 were inactivated or not altered while the other two branches of UPR were upregulated. These observations are of great interest as the activation of ATF6 is known to require ER-to-Golgi trafficking and proteolytic processing that involves both the ER and the Golgi apparatus [47]. In fact, co-localization of ATF6 and the Golgi was lower in the liver cells treated with the drugs and alcohol than in the cells treated with the specific ER stress inducing agent tunicamycin or thapsigargin [46]. In parallel to the reduction of ATF6 in the Golgi, marked Golgi fragmentation was observed in the RTV and LPV-treated liver cells (Figure 2), which was concentration and time dependent. Of note, the Golgi fragmentation was not due to mitosis as the drug-induced fragmentation was detected in both HepG2 cells and rarely dividing primary hepatocytes and liver tissues [46]. Apoptosis did not cause the fragmentation either as increased caspase activities were not detected until hours after the drug treatments and pancaspase inhibitors did not show any rescue effects on the drug-induced Golgi fragmentation. Moreover, variations in severity of the fragmentation were observed in response to other anti-HIV drugs including amprenavir, darunavir and nelfinavir, which were correlated with downstream ER stress and cell death and fatty liver injury [46]. All these pieces of evidence suggest that Golgi dysfunction and disturbed ER-Golgi trafficking contributes to the anti-HIV drugs and/or alcohol-induced liver disorders.

**Figure 3:**
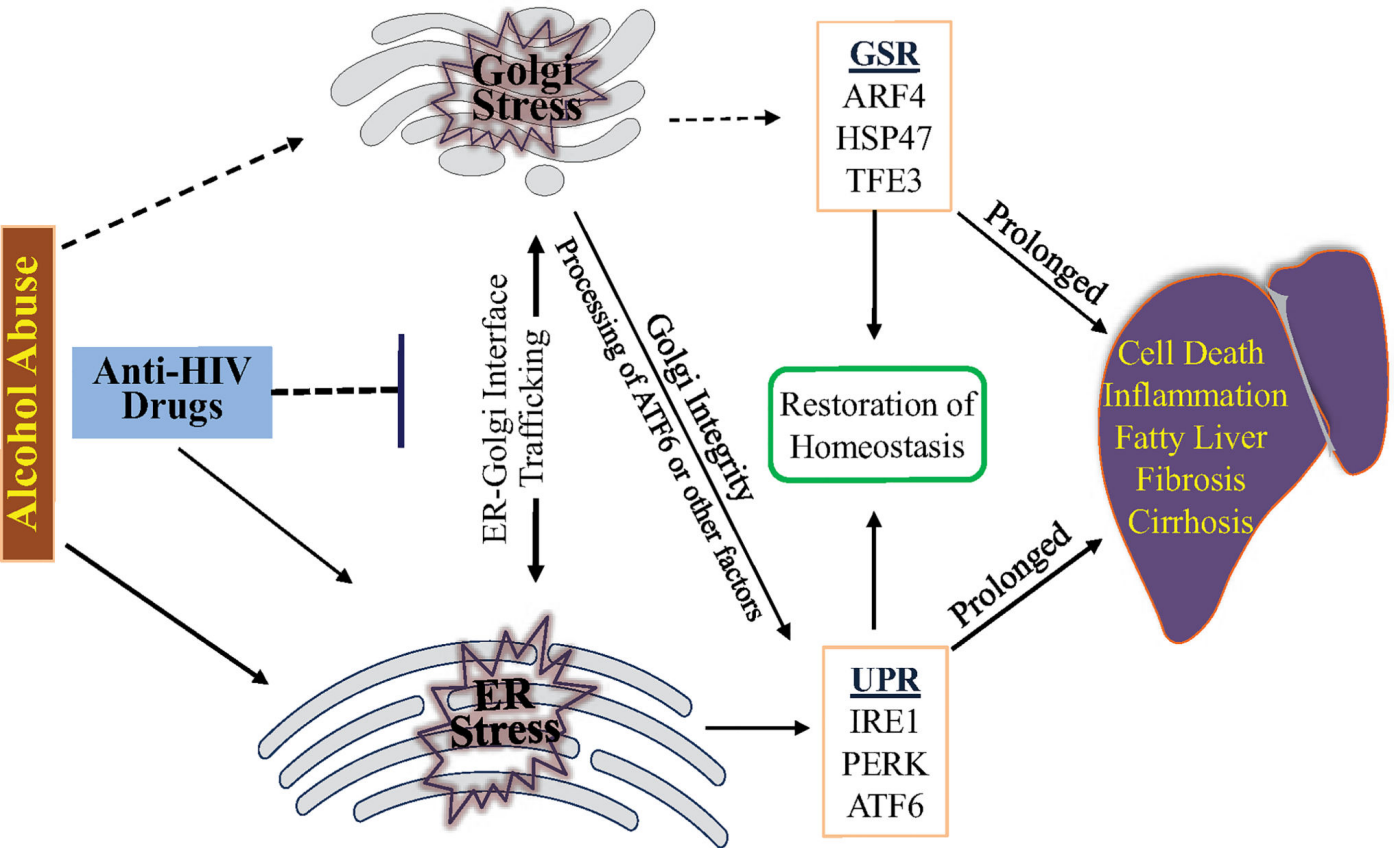
Working model on the role of disrupted ER-Golgi trafficking in anti-HIV drug and alcohol abuse-induced liver disorder. Anti-HIV drugs interfere with the ER-Golgi trafficking and compromise Golgi integrity causing Golgi stress and/or ER stress. The Golgi stress triggers Golgi stress response (GSR). GSR either restores homeostasis or induces injury under prolonged stress condition, the later of which can be worsened by alcohol-induced ER stress and unfolded protein response (UPR)

The ER-Golgi trafficking is bi-directional and responsible for biogenesis and intracellular distribution of biomolecules [48]. ER-to-Golgi trafficking or anterograde transportation mediated by the COPII complexes moves newly synthesized proteins and lipids to Golgi for processing, sorting and redistribution. Meanwhile, Golgi-to-ER trafficking or retrograde transportation mediated by the COPI complexes ensures recycling of lipids, fluids and ER escaped proteins [48,49]. Impairments of either anterograde or retrograde could eventually collapse the whole ER-Golgi trafficking and trigger cellular stress responses leading to injury [20,21, 46,48]. Thus, integrity of the ER-Golgi trafficking is essential for maintaining Golgi morphology [48–50]. The Golgi fragmentation observed by us indicates that the drugs stress the Golgi and disrupt the ER-Golgi trafficking. Indeed, Golgi stress response markers, GCP60 and HSP47 were increased in the drug-treated liver cells and knockdown of TFE3 worsened the drug-induced cell death [46]. Further, the anterograde ER-to-Golgi trafficking is likely affected more than the retrograde trafficking by the anti-HIV drugs and alcohol. This is because the effects of the drugs on Golgi fragmentation are similar to the effects of H89. H89 is a protein kinase A (PKA) inhibitor. PKA is required for the initiation and assembly of the COPII complexes [51]. In addition, BFA is known to inhibit specifically the assembly of COPI complexes [30] and its effects on the Golgi fragmentation are different from the effects of the anti-HIV drugs. Thus, it is conceivable that anti-HIV drugs interfere with the ER-Golgi trafficking and Golgi integrity causes Golgi stress and/or ER stress (Figure 3). The Golgi stress triggers Golgi stress response, which either restores Golgi homeostasis or induces injury depending on stress conditions. Alcohol consumption may deteriorate the drug-induced Golgi dysfunction and hepatic injury through interfering with the metabolism of the anti-HIV drugs as five to ten-fold increases of the drug concentrations were observed in the blood of animals fed alcohol [18,46].

## Perspectives

The primary target of antiviral drug and alcohol abuse appears to be the ER-Golgi trafficking based on our recent observations, which could explain a few aspects of pathological consequences in the hepatocytes that synthesize and secrete large amounts of substances. Disruptions in the ER-Golgi trafficking will have impact on the integrity and function of the Golgi apparatus. The stressed Golgi either respond to restore its homeostasis that potentially involves ARF4, HSP47 and TFE3 or dissemble to generate danger signals to stimulate the general immune response that eliminates the cells containing the damaged Golgi. The impaired ER-Golgi trafficking could also suspend processing and activation of transcription factors such as ATF6 that is involved in maintaining ER homeostasis. Loss of the ER homeostasis will stress the ER which is well established to induce cell death and disease development. Thus, vicious cycles resulted from the disrupted ER-Golgi trafficking most likely occurs in the liver cells that metabolize both the drugs and alcohol.

The question is what specific molecular components of the ER-Golgi traffic machineries are affected by the anti-HIV drugs and/or alcohol. The trafficking between ER and Golgi mediated by the endomembrane system involves vesicle budding, uncoating, docking and fusion that are often through recruitments of effectors such as vesicle tethers, SNAREs, membrane and motor proteins. These effectors are regulated by the Rab GTPases that belong to a large family of small GTP-binding proteins and accomplish their functions by switching between an inactive GDP bound and an active GTP-bound form. About one third of the 60 members of the Rab GTPase family found in human cells have been associated with either the ER or Golgi complex or the membrane intermediates at their interface. Since majority of the Rab proteins and effectors are not well characterized, it is hard to predict specific targets of the antiviral drugs. However, considering that the drugs were initially developed to prevent viral replication by selectively binding to viral *proteases* (e.g. HIV-1 *protease*) and blocking proteolytic cleavage of protein precursors required for the virus production, one would speculate that the drugs could also have some unintended effects on certain Rab proteins or effectors of the ER-Golgi trafficking machinery that require proteolytic cleavage for their maturation or functional activation. Future research should be directed to seek these unintended targets, which would provide molecular basis for modifications of the current anti-HIV drugs so that minimize the side effects of anti-HIV or other virus medicine.
